# Endocardial versus whole-myocardial tracking global longitudinal strain analysis in patients with hypertrophic cardiomyopathy: A preliminary comparative study

**DOI:** 10.1371/journal.pone.0288421

**Published:** 2023-07-11

**Authors:** Jiesuck Park, Yeonyee E. Yoon, Eun Ju Chun, Hong-Mi Choi, In-Chang Hwang, Hyun Jung Lee, Jun Bean Park, Seung Pyo Lee, Hyung Kwan Kim, Yong Jin Kim, Goo-Yeong Cho

**Affiliations:** 1 Department of Cardiology, Cardiovascular Center, Seoul National University Bundang Hospital, Seongnam, Gyeonggi-do, Republic of Korea; 2 Department of Internal Medicine, Seoul National University College of Medicine, Seoul, Republic of Korea; 3 Department of Radiology, Cardiovascular Center, Seoul National University Bundang Hospital, Seongnam, Gyeonggi-do, Republic of Korea; 4 Department of Radiology, Seoul National University College of Medicine, Seoul, Republic of Korea; 5 Department of Internal Medicine and Cardiovascular Center, Seoul National University Hospital, Seoul, Republic of Korea; Oswaldo Cruz Foundation, BRAZIL

## Abstract

**Background and objectives:**

We investigated whether the feasibility of left ventricular (LV) global longitudinal strain (GLS) in hypertrophic cardiomyopathy (HCM) varies according to the methodology (e.g. endocardial vs. whole myocardial tracking techniques).

**Methods:**

We retrospectively analyzed 111 consecutive patients with HCM (median age, 58 years; male, 68.5%) who underwent both transthoracic echocardiography (TTE) and cardiac magnetic resonance imaging (apical 29.7%, septal 33.3%, and diffuse or mixed 37.0%). TTE-whole myocardial and TTE-endocardial GLS were measured and compared in terms of association with late gadolinium enhancement (LGE) extent and discrimination performance for extensive LGE (>15% of the LV myocardium).

**Results:**

Although TTE-whole myocardial and TTE-endocardial GLS were significantly correlated, absolute TTE-endocardial GLS values (19.3 [16.2–21.9] %) were higher than TTE-whole myocardial GLS values (13.3[10.9–15.6] %, p<0.001). Both TTE-derived GLS parameters were significantly correlated with the LGE extent and independently associated with extensive LGE (odds ratio [OR] 1.30, p = 0.022; and OR 1.24, p = 0.013, respectively). Discrimination performance for extensive LGE was comparable between TTE-whole myocardial and TTE-endocardial GLS (area under the curve [AUC], 0.747 and 0.754, respectively, p_difference_ = 0.610). However, among patients with higher LV mass index (>70 g/m^2^), only TTE-whole myocardial GLS correlated with LGE extent and was independently associated with extensive LGE (OR 1.35, p = 0.042), while TTE-endocardial GLS did not. Additionally, TTE-whole myocardial GLS had better discrimination performance for extensive LGE than TTE-endocardial GLS (AUC, 0.705 and 0.668, respectively, p_difference_ = 0.006).

**Conclusion:**

TTE-derived GLS using either the endocardial or whole myocardial tracking technique is feasible in patients with HCM. However, in those with severe hypertrophy, TTE-whole myocardial GLS is better than TTE-endocardial GLS.

## Introduction

Left ventricular (LV) global longitudinal strain (GLS) analysis is widely utilized to assess myocardial deformation [1]. The LV GLS is not only useful as a measure of LV systolic dysfunction; its clinical value has also been demonstrated for early diagnosis, risk stratification, and determining the long-term prognosis in many cardiovascular diseases, including ischemic heart disease, heart failure, valvular heart disease, and various cardiomyopathies [2]. Impairment in the LV GLS also shows clinical association with metabolic derangement of the myocardium, leading to LV remodeling and tissue fibrosis [3, 4].

While advancements in speckle tracking echocardiography have enabled the wide availability of strain assessment in clinical practice [2], competitive methodologies exist, resulting in variability in GLS assessment [5]. For example, despite standardization efforts, vendors still vary in the myocardial layers tracked to generate the GLS; the most common methods involve tracking the LV endocardial border (endocardial GLS) or the whole myocardium (whole myocardial GLS). Although data regarding the potential advantages and disadvantages of various GLS analysis algorithms, and the appropriate clinical indications, are relatively scarce, previous studies suggest that the endocardial GLS has advantages as a sensitive indicator of myocardial injury primarily affecting the endocardium, as observed in patients with myocardial ischemia and infarction [6–8]. However, in patients with thickened myocardium, such as those with hypertrophic cardiomyopathy (HCM), myocardial injury does not occur in an endocardial-dominant manner. Thus, it is unclear whether the endocardial GLS retains an advantage over the whole myocardial GLS as a feasible and sensitive parameter for identifying LV dysfunction and myocardial injury in such patients.

Therefore, in the present study, we measured both LV endocardial and whole myocardial GLS on transthoracic echocardiography (TTE) in patients with HCM, and compared these parameters against GLS on cardiac magnetic resonance imaging (CMR) in terms of the association with the extent of myocardial fibrosis. In addition, we evaluated the discrimination performance of these GLS parameters for the detection of extensive late gadolinium enhancement (LGE), and determined whether the performance differed according to a high LV mass index (LVMI).

## Materials and methods

### Study population

We retrospectively screened 306 consecutive patients with HCM who underwent CMR, including both cine- and LGE-CMR, at Seoul National University Bundang Hospital, between 2010 and 2019. The clinical diagnosis of HCM was established by confirming a maximal end-diastolic wall thickness of ≥15 mm at any site of the LV, with an absence of other causes of hypertrophy [9]. Among these, 116 patients were selected who underwent both TTE and CMR within 6 months. After excluding cases without adequate TTE or CMR images for the assessment of LV GLS (n = 4; three TTE cases and one CMR case) and LGE extent of the LV myocardium (n = 1), a total of 111 patients was included as the final study population. The institutional review board of Seoul National University Bundang Hospital approved the study protocol (IRB No. B-1612-376-105) and waived the requirement for informed consent due to the retrospective nature of the study design. All the clinical information was fully anonymized before data analysis. The study was conducted in compliance with the Declaration of Helsinki 2013.

### TTE and strain analysis

**Fig 1:**
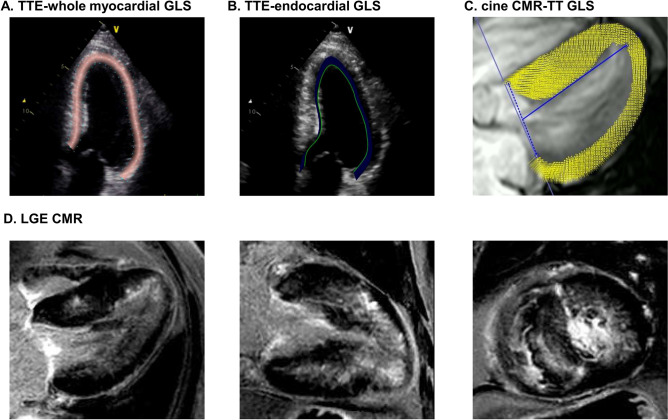
Representative case. LV GLS was measured on TTE using whole-myocardial tracking (11.3%) (A) or endocardial tracking (19.9%) (B), and on CMR using TT (6.6%) (C). LGE-CMR revealed extensive LGE, with an LGE extent of 43.0% (D).

Echocardiography images were obtained using a standard ultrasound machine (Vivid 7, E9 and E95 ultrasound system; GE Vingmed Ultrasound AS, Horten, Norway) at a frame rate ranging from 50 to 80 Hertz with a 2.5-MHz probe. Following guideline recommendations, 2-dimensional, M-mode, and Doppler images were acquired using a standard protocol [10, 11]. LV end-systolic and end-diastolic volumes were measured to calculate the LV ejection fraction (LVEF), using the Simpson biplane method, from apical 4- and 2-chamber views. Using tissue Doppler images, the early mitral annular velocity (e’) was measured at the septal side of the mitral annulus. An image specialist, blinded to clinical data, performed the strain analysis using two different dedicated software programs: one that tracked the endocardial border (TomTec Image Arena 4.6, Munich, Germany) and one that tracked the whole myocardium (EchoPAC PC BT20, GE Medical Systems, Horten, Norway) (Fig 1). TTE-endocardial and TTE-whole myocardial GLS were calculated using the same TTE image frames focused on LV. All the echocardiography images were saved as DICOM files and were send to the corresponding workstations for strain measures.

For TTE-endocardial GLS assessment, the LV endocardial border was delineated semi-automatically at the end-systolic frame on apical view images using TomTec software. Subsequently, the software automatically tracked echo-speckles along the endocardial border over a single cardiac cycle. Peak longitudinal systolic strain in each myocardial segment was captured as the peak negative value during the cardiac cycle. Subsequently, the longitudinal strain of the selected image plane was calculated as the average of the strain values of the myocardial segments. The final LV endocardial GLS was calculated as the mean value of the longitudinal strain derived from apical 4-, 3-, and 2-chamber view images.

For TTE-whole myocardial GLS assessment, the region of interest was determined automatically and manually adjusted to fit the thickness of the myocardium using EchoPac software. As with the TTE-endocardial GLS, the longitudinal strain was calculated in each standard apical view over a single cardiac cycle, based on the peak strain values of the myocardial segments. The calculated longitudinal strain values were then averaged to derive the LV whole myocardial GLS.

Since LV GLS represents longitudinal shortening of myocardial fibers, it is expressed as negative values; thus, negative LV GLS values were converted to absolute |x| values for simpler interpretation. LV GLS values closer to 0 indicate more impaired LV systolic function.

### CMR LV strain and LGE extent analysis

Details regarding CMR LV strain the LGE extent analysis are provided in the S1 Appendix. As with TTE-derived GLS values, the peak negative value (peak systolic strain) on was converted to an absolute |x| value, designated as the CMR-TT GLS (Fig 1) [12]. Furthermore, extensive LGE was defined as an LGE extent >15% of the LV mass, which has been reported to be associated with an increased risk of sudden cardiac death [13].

### Statistical analysis

Baseline clinical characteristics are presented as the median with interquartile range for continuous variables and as the number with percentage for categorical variables. Differences between GLS parameters derived from different software algorithms and imaging modalities were evaluated using the paired sample t-test. Spearman’s correlation analysis was used to investigate correlations between TTE-derived GLS values and CMR parameters, including CMR-TT GLS, LVMI, and LGE extent. Agreement between TTE-derived GLS and CMR-TT GLS values were assessed using the Bland-Altman plot. Discrimination performance for the presence of extensive LGE was assessed and compared between different GLS parameters using the area under the receiver operating characteristic (AUROC) curve, with 95% confidence intervals (CIs). In addition, logistic regression modelling was used to estimated odds ratios (ORs) of clinical and TTE parameters for extensive LGE. ORs of TTE-derived GLS values were also estimated with adjustment for age, sex, systolic blood pressure, and echocardiographic parameters (LVEF, maximal LV wall thickness, LA volume index, and e’ velocity). Additionally, we performed a subgroup analysis to evaluate whether the discrimination performance for, and association with, extensive LGE differed in patients with high LVMI; the median LVMI value (70 g/m^2^) was used as the cutoff value.

The incremental predictive value of TTE-derived GLS was evaluated by multivariate logistic regression modeling with the following sequences: Model 1 included age, sex, systolic blood pressure, LVEF, maximal LV wall thickness, LA volume index, and e’ velocity; Model 2 added TTE-whole myocardial GLS to Model 1; and Model 3 added TTE-endocardial GLS to Model 1. Model discriminatory performance was assessed by the AUROC curve. The differences in AUROCs were compared with the DeLong test.

Furthermore, we categorized patients according to the optimal cutoff values for discriminating extensive LGE on TTE-derived GLS parameters to evaluate discrepancies between TTE-derived GLS parameters. The optimal cutoff value of each GLS parameter was determined using the receiver operating characteristics curves for extensive LGE, based on the Youden index [14]. After classification, the number of patients with extensive LGE and the LGE extent were compared between groups.

All statistical analyses were performed using R software, V.4.1.1 (R Development Core Team, Vienna, Austria). Two-sided p-values <0.05 were considered statistically significant.

## Results

### Baseline characteristics

Clinical characteristics of the 111 patients (age, 58 [49–68] years; male, 68.5%) are summarized in Table 1. Apical, septal, and diffuse or mixed type HCM was present in 29.7%, 33.3%, and 37.0% of patients, respectively. On CMR, the median LVMI was 70 (58–96) g/m^2^. LGE was found in 110 (99.1%) patients, with extensive LGE (>15% of the LV mass) in 16 (14.5%) patients. The median interval between CMR and TTE acquisition was 15 (5–31) days.

**Table 1:** Baseline characteristics.

	Total population (n = 111)
Age, years	58 (49–68)
Male, n (%)	76 (68.5)
Body mass index, kg/m^2^	25 (24–28)
HCM type, n (%)	
Apical	33 (29.7)
Septal	37 (33.3)
Diffuse	8 (7.2)
Mixed	33 (29.7)
***TTE parameters***	
LV end-diastolic volume, ml	75 (58–84)
LV end-systolic volume, ml	25 (20–30)
LV ejection fraction, %	66 (61–69)
LV ejection fraction ≥50%, n (%)	110 (99.1)
Maximal wall thickness, mm	18 (16–20)
E velocity, m/s	0.6 (0.6–0.7)
A velocity, m/s	0.7 (0.6–0.9)
s’ velocity, cm/s	7.0 (5.8–8.1)
e’ velocity, cm/s	4.9 (4.2–6.1)
a’ velocity, cm/s	7.5 (6.4–9.1)
E/e’ ratio	12.9 (9.6–15.6)
Whole myocardial GLS, %	13.3 (10.9–15.6)
Endocardial GLS, %	19.3 (16.2–21.9)
***CMR parameters***	
LV end-diastolic volume, ml	112 (95–126)
LV end-systolic volume, ml	33 (26–44)
LV ejection fraction, %	69 (62–74)
LV mass index, g/m^2^	70 (58–96)
Tissue tracking GLS, %	10.9 (9.4–12.6)
LGE mass, g	5.0 (2.4–15.6)
LGE extent, %	4.5 (2.2–11.6)
Extensive LGE (> 15% of LV mass), n (%)	16 (14.4)

Data are presented as the median (interquartile range) for continuous variables and number (percentage) for categorical variables

Abbreviations: CMR, cardiovascular magnetic resonance imaging; ***GLS***, global longitudinal strain; HCM, hypertrophic cardiomyopathy; LGE, late gadolinium enhancement; LV, left ventricle; TTE, transthoracic echocardiography

### Correlations between GLS with CMR parameters

**Fig 2:**
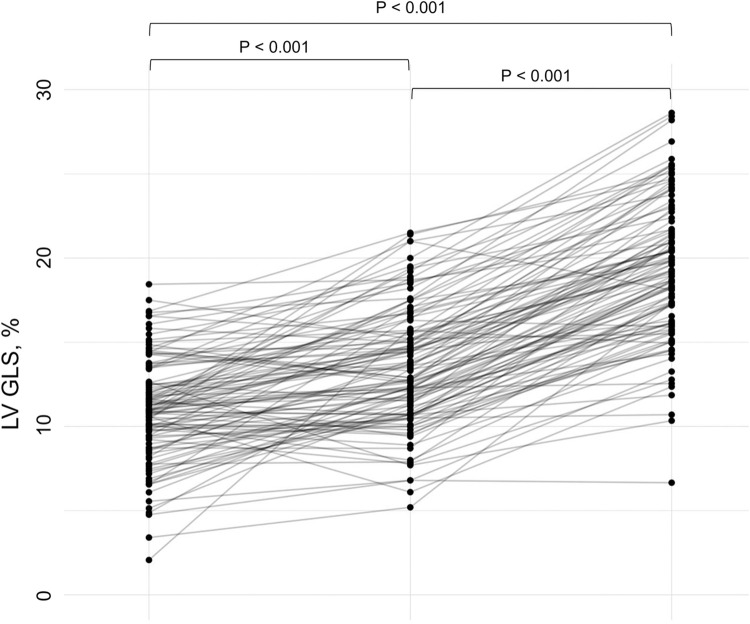
GLS values derived from different algorithms and modalities. TTE-endocardial and TTE-whole myocardial GLS values were significantly higher than CMR-TT GLS values. Additionally, TTE-endocardial GLS values were significantly higher than TTE-whole myocardial GLS values.

The assessment of LV GLS on TTE and CMR images using different software algorithms in a representative case is shown in Fig 1. TTE-endocardial GLS values (19.3 [16.2–21.9] %) were significantly higher than TTE-whole myocardial (13.3 [10.9–15.6] %, p<0.001) and CMR-TT GLS values (10.9 [9.4–12.6] %, p<0.001) (Fig 2). TTE-endocardial and TTE-whole myocardial GLS were significantly correlated (r = 0.718, p<0.001) (S1 Fig). Although both TTE-based GLS parameters demonstrated moderate correlations with CMR-TT GLS, TTE-whole myocardial GLS showed a relatively stronger correlation (r = 0.553, p<0.001) than TTE-endocardial GLS (r = 0.442, p<0.001) (S1 Fig). Bland-Altman plots demonstrated poor agreement between TTE-endocardial and CMR-TT GLS, which was more prominent than that between TTE-whole myocardial and CMR-TT GLS (S2 Fig).

**Fig 3:**
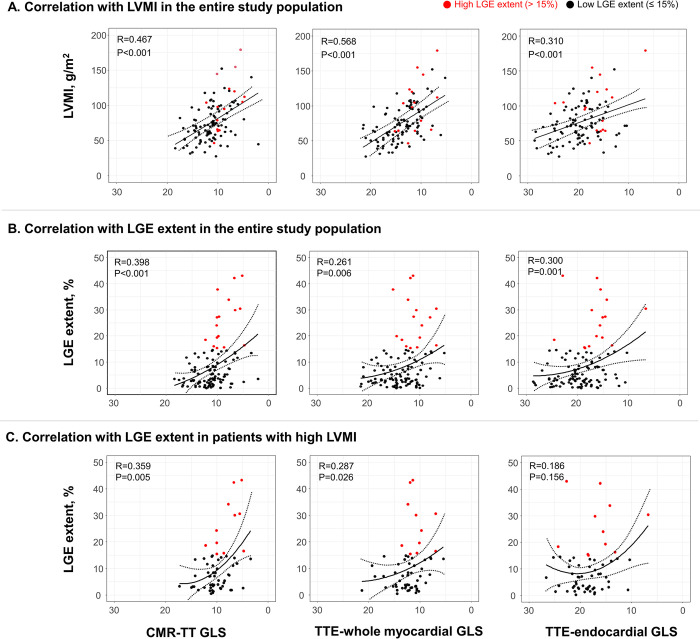
Correlations between GLS parameters and LVMI and LGE extent. Each GLS parameter demonstrated a significant correlation with LVMI; however, the strongest correlation was obtained with TTE-whole myocardial GLS (A). LGE extent showed a relatively weak, but significant correlation with each GLS parameter (B). However, in the subgroup of patients with high LVMI, CMR-TT GLS and TTE-whole myocardial GLS, but not TTE-endocardial GLS, were significantly correlated with LGE extent (C).

Results of the correlational analyses of each GLS parameter with LVMI and LGE extent are shown in Fig 3. All GLS parameters showed significant correlation with LVMI; the strongest correlation was obtained with TTE-whole myocardial GLS (r = 0.568, p<0.001) followed by CMR-TT GLS (r = 0.467, p<0.001) and TTE-endocardial GLS (r = 0.310, p<0.001) (Fig 3A). All GLS parameters also showed significant, but relatively weak, correlation with LGE extent (CMR-TT GLS: r = 0.398, p<0.001; TTE-whole myocardial GLS: r = 0.261, p = 0.006; and TTE-endocardial GLS: r = 0.300, p = 0.001) (Fig 3B). Interestingly, in the subgroup analysis among patients with high LVMI, significant correlation with LGE extent was observed for CMR-TT GLS (r = 0. 359, p<0.001) and TTE-whole myocardial GLS (r = 0.287, p = 0.026), but not for TTE-endocardial GLS (r = 0.186, p = 0.156) (Fig 3C).

### Discrimination performance of different GLS parameters for extensive LGE

On ROC curve analysis, discrimination performance for extensive LGE was comparable between TTE-whole myocardial GLS (AUROC 0.747, 95% CI 0.727–0.767) and TTE-endocardial GLS (AUROC 0.754, 95% CI 0.729–0.779; p_difference_ = 0.610), although both parameters showed lower performance than CMR-TT GLS (AUROC 0.781, 95% CI 0.762–0.779; p_difference_ = 0.001 for TTE-whole myocardial GLS; p_difference_ = 0.027 for TTE-endocardial GLS) (S3A Fig). However, in the subgroup with high LVMI, although CMR-TT GLS still showed the best discrimination performance for extensive LGE (AUROC 0.755, 95% CI 0.728–783), there was a significant difference in performance between TTE-whole myocardial and TTE-endocardial GLS; TTE-whole myocardial GLS showed better discrimination performance for extensive LGE (AUROC 0.705, 95% CI, 0.679–0.731) than TTE-endocardial GLS (AUROC 0.688, 95% CI, 0.634–0.701; P_difference_ = 0.006) (S3B Fig).

### Independent and incremental predictive value of TTE-derived GLS parameters for extensive LGE

On univariate logistic regression analysis for extensive LGE, e’ velocity (unadjusted OR 1.64, 65% CI 1.10–2.61, p = 0.023), TTE-whole myocardial GLS (unadjusted OR 1.32, 95% CI 1.11–1.62, p = 0.004), and TTE-endocardial GLS (unadjusted OR 1.25, 95% CI 1.08–1.47, p = 0.004) were significantly associated with extensive LGE (Table 2). On multivariate analysis, both TTE-whole myocardial and TTE-endocardial GLS parameters maintained independent association with extensive LGE (adjusted OR 1.30, 95% CI 1.05–1.66, p = 0.022; and adjusted OR 1.24, 95% CI 1.06–1.50, p = 0.013, respectively), even after adjustment for clinical and other TTE parameters (Table 2). In the subgroup with high LVMI, only TTE-whole myocardial GLS showed significant and independent association with extensive LGE (unadjusted OR 1.31, 95% CI 1.02–1.78, p = 0.031; and adjusted OR 1.35, 95% CI 1.01–1.98, p = 0.042), while TTE-endocardial GLS did not (unadjusted OR 1.17, 95% CI 0.99–1.42, p = 0.076; and adjusted OR 1.19, 95% CI 0.97–1.49, p = 0.107) (Table 3).

**Table 2:** Independent associations of clinical and echocardiographic parameters with extensive LGE.

	Univariate OR (95% CI)	p-value	Multivariate OR^a^ (95% CI)	p-value	Multivariate OR^a^ (95% CI)	p-value
Age, per 10-year increase	0.96 (0.65–1.43)	0.833	0.79 (0.45–1.36)	0.403	0.48 (0.27–2.49)	0.499
Male	1.45 (0.46–5.53)	0.545	0.92 (0.22–4.26)	0.907	1.85 (0.47–9.18)	0.413
Systolic blood pressure, per 10-mmHg increase	0.78 (0.55–1.05)	0.126	0.66 (0.43–0.93)	0.035	0.69 (0.45–0.96)	0.047
LVEF, per 10% decrease	1.87 (0.81–4.43)	0.135	1.13 (0.37–3.87)	0.838	1.16 (0.39–3.85)	0.799
Maximal wall thickness, per 1-mm increase	1.10 (0.96–1.25)	0.153	1.00 (0.83–1.21)	0.991	1.03 (0.85–1.24)	0.764
LAVI, per 10-ml/m^2^ increase	1.19 (0.85–1.63)	0.280	1.17 (0.81–1.71)	0.409	1.08 (0.74–1.58)	0.672
e’ velocity, per 1-cm/s decrease	1.64 (1.10–2.61)	0.023	1.58 (0.93–2.91)	0.111	1.76 (1.04–3.28)	0.042
TTE-whole myocardial GLS, per 1% decrease	1.32 (1.11–1.62)	0.004	1.30 (1.05–1.66)	0.022	-	-
TTE-endocardial GLS, per 1% decrease	1.25 (1.08–1.47)	0.004	-	-	1.24 (1.06–1.50)	0.013

^a^Adjusted for age, sex, systolic blood pressure, LVEF, maximal LV wall thickness, left atrial volume index, and e’ velocity

Abbreviation: CI, confidence interval; GLS, global longitudinal strain; LAVI, left atrial volume index; LGE, late gadolinium enhancement; LVEF, left ventricular ejection fraction; OR, odds ratio; TTE, transthoracic echocardiography

**Table 3:** Independent predictors of extensive LGE in patients with high LVMI.

	Univariate OR (95% CI)	p-value	Multivariate OR^a^ (95% CI)	p-value	Multivariate OR^a^ (95% CI)	p-value
Age, per 10-year increase	1.19 (0.77–1.90)	0.454	1.48 (0.75–3.02)	0.255	1.48 (0.75–3.03)	0.260
Male	1.46 (0.32–10.45)	0.656	2.47 (0.33–2.83)	0.408	4.08 (0.52–5.36)	0.218
Systolic blood pressure, per 10-mmHg increase	0.83 (0.54–1.19)	0.354	0.74 (0.45–1.10)	0.179	0.80 (0.51–1.17)	0.291
LVEF, per 10% decrease	1.81 (0.68–5.11)	0.223	0.75 (0.19–2.99)	0.659	0.79 (0.21–2.97)	0.714
Maximal wall thickness, per 1-mm increase	1.08 (0.91–1.26)	0.354	1.09 (0.86–1.39)	0.446	1.12 (0.89–1.42)	0.321
LAVI, per 10-ml/m^2^ increase	1.22 (0.82–1.79)	0.304	1.21 (0.80–1.87)	0.359	1.15 (0.76–1.76)	0.491
e’ velocity, per 1-cm/s decrease	1.29 (0.81–2.20)	0.316	1.15 (0.59–2.28)	0.684	1.27 (0.68–2.54)	0.464
TTE-whole myocardial GLS, per 1% decrease	1.31 (1.02–1.78)	0.031	1.35 (1.01–1.98)	0.042	-	-
TTE-endocardial GLS, per 1% decrease	1.17 (0.99–1.42)	0.076	-	-	1.19 (0.97–1.49)	0.107

^a^Adjusted for age, sex, systolic blood pressure, LVEF, maximal LV wall thickness, left atrial volume index, and e’ velocity

Abbreviation: CI, confidence interval; GLS, global longitudinal strain; LAVI, left atrial volume index; LGE, late gadolinium enhancement; LVEF, left ventricular ejection fraction; OR, odds ratio; TTE, transthoracic echocardiography

In the prediction models for extensive LGE comprising clinical (age, sex, and systolic blood pressure) and TTE parameters (LVEF, maximal wall thickness, left atrial volume index [LAVI], and e’ velocity) in the entire study population, the addition of TTE-whole myocardial GLS to the model improved the predictive value (AUROC 0.767 vs. 0.809, P_difference_ = 0.001); similarly, the addition of TTE-endocardial GLS to the model improved the predictive value (AUROC 0.767 vs. 0.813, P_difference_ = 0.001) (Table 4). However, in the subgroup with high LVMI, the addition of TTE-whole myocardial GLS to the model improved the predictive value (AUROC 0.694 vs. 0.771, P_difference_ = 0.001); however, the addition of TTE-endocardial GLS to the model did not improve the predictive value (AUROC 0.694 vs. 0.717, P_difference_ = 0.133) (Table 4).

**Table 4:** Incremental predictive value of TTE-derived GLS parameters for extensive LGE.

		Total population		Subgroup with high LVMI	
		AUROC (95% CI)	p_difference_	AUROC (95% CI)	p_difference_
Model 1	Clinical + TTE parameters^a^	0.767 (0.746–0.788)	Reference	0.694 (0.661–0.726)	reference
Model 2	Model 1 + TTE-whole myocardial GLS	0.809 (0.792–0.826)	0.001	0.771 (0.745–0.797)	0.001
Model 3	Model 1 + TTE-endocardial GLS	0.813 (0.790–0.836)	0.001	0.717 (0.685–0.748)	0.133

^a^Adjusted for age, sex, systolic blood pressure, LVEF, maximal LV wall thickness, left atrial volume index, and e’ velocity

Abbreviation: AUROC, area under the receiver operating characteristic; CI, confidence interval; GLS, lobal longitudinal strain; LVEF, left ventricular ejection fraction; LVMI, left ventricular mass index; TTE, transthoracic echocardiography

### Discrepancies between TTE-whole myocardial and TTE-endocardial GLS

**Fig 4:**
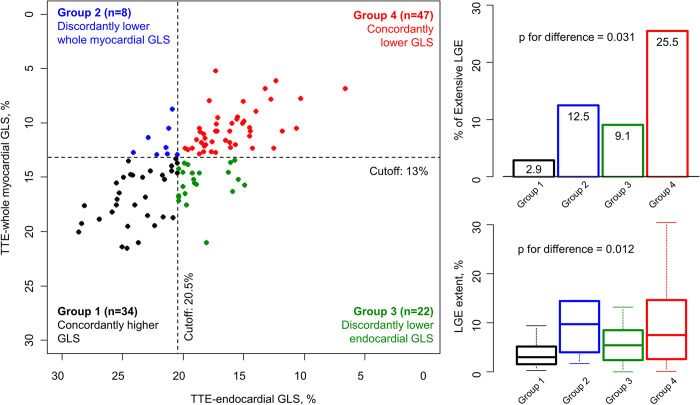
Discrepancies between echocardiographic strain parameters derived from different tracking algorithms. Patients were stratified according to discrepancy between TTE-whole myocardial and TTE-endocardial GLS, using the corresponding optimal cutoff values for detecting extensive LGE (13.0% and 20.5%, respectively). Among the four patient groups, the rate of extensive LGE was highest in Group 4 (concordantly low GLS). Compared to Group 2 (discordantly lower TTE-whole myocardial GLS), Group 3 (discordantly lower TTE-endocardial GLS) showed a higher rate of extensive LGE. Consistent trends were also observed for LGE extent.

To evaluate discrepancies between TTE-whole myocardial and TTE-endocardial GLS, the optimal cutoff values for discriminating extensive LGE were utilized to categorize patients with HCM. Notably, the optimal cutoff value for TTE-whole myocardial GLS (13.0%) was comparable to that for CMR-TT GLS (11.0%), but was much smaller than that for TTE-endocardial GLS (20.5%). Patients were categorized into Group 1 (n = 34, 30.6%), with concordantly preserved GLS; Group 2 (n = 8, 7.2%), with impaired TTE-whole myocardial GLS, but preserved TTE-endocardial GLS; Group 3 (n = 22, 19.8%), with preserved TTE-whole myocardial GLS, but impaired TTE-endocardial GLS; and Group 4 (n = 47, 42.3%), with concordantly impaired GLS (Fig 4). Extensive LGE was most commonly observed in Group 4 (12 of 47, 25.5%), but was rarely observed in Group 1 (1 of 34, 2.9%). Interestingly, among discordant groups, extensive LGE was more commonly observed in Group 2 (1 of 8, 12.5%) than in Group 3 (2 of 22, 9.1%). Comparisons in LGE extent between groups showed a similar pattern (Fig 4). Results of comparisons between groups for other TTE and CMR parameters are provided in S1 Table. Similar to that for LGE extent, LVMI was higher in Group 2 than in Group 3, and s’ was lower in Group 2 than in Group 3.

## Discussion

In the current study, we assessed the feasibility of LV GLS derived from different myocardial layers–the endocardial border and the whole myocardium–in patients with HCM. In the entire study population, both TTE-whole myocardial and TTE-endocardial GLS were significantly correlated with CMR-derived GLS, LVMI, and LGE extent, and were independently associated with extensive LGE over clinical and echocardiographic features. However, among patients with HCM with a high LVMI, TTE-whole myocardial GLS, but not TTE-endocardial GLS, was significantly correlated with the LGE extent and was independently associated with extensive LGE. The discrimination performance for extensive LGE was comparable for TTE-derived GLS parameters in the entire study population, but was significantly better with TTE-whole myocardial GLS than with TTE-endocardial GLS in patients with high LVMI. In cases with discrepancy between TTE-whole myocardial and TTE-endocardial GLS, impaired TTE-whole myocardial GLS better reflected the presence of extensive LGE and greater LGE extent.

Although most prototypical vendors are currently evolving towards a comprehensive layer-specific strain analysis, these have been mainly applied for research purposes. Therefore, we considered comparisons in the feasibility and performance of the two most commonly used GLS algorithms as clinically relevant, especially in patients with HCM with thickened myocardium. In the present study, despite significant correlations between TTE-based GLS parameters, the absolute values of TTE-endocardial GLS were significantly higher than those of TTE-whole myocardial GLS. Given a transmural gradient in GLS (from high absolute strain values in the endocardium to low values in the epicardium) that exists in the healthy population [15, 16], this finding is not surprising. Furthermore, it is also known that this trend is augmented in patients with HCM, resulting in a higher ratio of endo- to epicardial GLS [15]. A greater endo- to epicardial GLS gradient is significantly associated with the maximal LV wall thickness and septal thickness, as well as with impaired LV diastolic function [15–17]. Therefore, although the current study did not compare endocardial GLS to epicardial GLS, the observation of higher absolute values for TTE-endocardial GLS than for TTE-whole myocardial GLS is reasonable. Importantly, in the present study, we found that TTE-endocardial GLS demonstrated higher variability and relatively weaker correlation with the LVMI compared to those for TTE-whole myocardial GLS. Since these results suggest that TTE-endocardial GLS might be less feasible in patients with HCM with severe LV hypertrophy, we additionally conducted subgroup analyses in patients with higher LVMI.

Although the association between TTE-derived GLS and myocardial fibrosis has been repeatedly demonstrated [18–21]. very few studies have attempted to compare the relationship between GLS and myocardial scar extent according to the myocardial layer where GLS was measured. We could find only one study that compared TTE-derived layer-specific GLS in patients with HCM [22]. In this previous study, the whole myocardial longitudinal strain in the hypertrophic area showed the best correlation with myocardial fibrosis in the hypertrophic area. Although this previous study mainly focused on regional strain assessment in the hypertrophic myocardium rather than the GLS, it provides some important clues on the advantage of TTE-whole myocardial GLS in patients with HCM. To the best of our knowledge, the current study is the first to include a head-to-head comparison between TTE-endocardial and TTE-whole myocardial GLS for the detection of extensive LGE in patients with HCM.

Although previous studies support endocardial GLS as a sensitive indicator of myocardial ischemia and infarction, which primarily affect the endomyocardium [6–8], there are concerns that the endocardial strain may not sensitively reflect myocardial change in patients with increased LV wall thickness [23]. Especially, in patients with HCM with thickened myocardium and increased LV mass, myocardial injury occurs in a non-endocardial dominant manner, leading to diffuse or patch-form LGE distribution [24]. Unlike that in the entire study population, TTE-endocardial GLS was not significantly correlated with the LGE extent and showed worse discrimination performance, and failed to show independent and incremental predictive value, for extensive LGE than TTE-whole myocardial GLS in the subgroup with higher LVMI. These results indicate that GLS assessment by the endocardial tracking technique might be limited as indicator of the extent of myocardial fibrosis, especially in those with severe hypertrophy. Furthermore, when a discrepancy exists between TTE-endocardial and TTE-whole myocardial GLS in patients with HCM, TTE-whole myocardial GLS would be a better choice for an accurate evaluation of global LV dysfunction and myocardial fibrosis. Therefore, although TTE-endocardial GLS is generally feasible in patients with HCM, caution may be warranted for its application.

The strength of the present study arises from its unique design, which evaluated three different GLS parameters at the patient level, derived from TTE and CMR images. Additionally, the present study findings provide practical value in the evaluation of patients with HCM via GLS assessment. However, the results should be interpreted under consideration of the following limitations. First, the study cohort was not population-based and comprised a relatively small number of patients from a single tertiary medical center. Although we screened consecutive patients with HCM, the inclusion criteria of TTE and CMR within 6 months inevitably limited the study population. Therefore, caution must be taken when applying the current preliminary results to the general population. In addition, our findings may have insufficient power to support firm conclusions regarding the discrepancy between TTE-whole myocardial and TTE-endocardial GLS. Especially for patients with high LVMI, the multivariate model could be exposed at risk of overfitting due to the relatively small number of cases. Secondly, the median time interval between CMR and TTE examinations was only 15 days; although small, this time gap might have differentially affected the correlations between GLS parameters and LGE extent, considering differences in examination conditions. Finally, the discrimination performance of TTE-derived GLS parameters for extensive LGE may vary in patients with HCM with higher LVMI [13, 25] or lower LGE extent [25]. Therefore, the cutoff values obtained in the present study should be applied with discretion in other study populations.

## Conclusions

GLS analysis on strain echocardiography using either the endocardial or whole myocardial tracking technique is feasible in patients with HCM. However, in those with increased LV mass, whole myocardial GLS is better than endocardial GLS for the assessment of global LV function and myocardial fibrosis.

## Supporting information

S1 Appendix CMR acquisition and analysis. (PDF)

S1 Table Echocardiographic and CMR features according to TTE-GLS discrepancy group. (PDF)

S1 Fig Plots of the correlation between each evaluated LV GLS parameter and LGE extent. (PDF)

S2 Fig Bland-Altman plots of the three evaluated LV GLS parameters. (PDF)

S3 Fig Discrimination performance of GLS parameters for extensive LGE. (PDF)

S1 Graphical abstract (TIF)
