# Supplementary material for: Endocardial versus whole-myocardial tracking global longitudinal strain analysis in patients with hypertrophic cardiomyopathy: A preliminary comparative study
Source: PLoS One. 2023 Jul 11;18(7):e0288421. doi: 10.1371/journal.pone.0288421 (PMC10335699; doi:10.1371/journal.pone.0288421)
Supplement: S1 Appendix — (PDF) [file pone.0288421.s001.pdf]

S1 Appendix. CMR acquisition and analysis

CMR images were acquired on a 1.5-T (Intera CV release 10; Philips Healthcare, Amsterdam, The Netherlands) or 3-T CMR unit (Philips Ingenia, Healthcare, Best, The Netherlands), with dedicated phased-array surface coils. All CMR images were obtained under electrocardiographic gating and breath-hold conditions. Steady-state free-precession (SSFP) cine-CMR images were obtained in the horizontal long axis, vertical long axis, and LV outflow tract. A short-axis stack view of the whole LV was obtained for regional wall motion and systolic function analysis. The cine-image sequence was acquired at 25-30 frames/R-R interval. Cine-CMR parameters were as follows: field of view, 320-370 mm; repetition time/echo time, 3.0-3.6/1.5-1.8 ms; flip angle, 45-60°; and slice thickness, 6-8 mm. LGE images were obtained 10 minutes after the intravenous administration of 0.2 mmol/kg of gadodiamide (Omniscan; GE Healthcare, Princeton, NJ, USA), using a phase-sensitive inversion-recovery turbo field echo sequence (repetition time/echo time, 4.5-4.6/1.3-1.5 ms; flip angle, 20-25°; and slice thickness, 8 mm).

Acquired CMR images were transferred into commercially available software (CVI42 5.10, Circle Cardiovascular Imaging, Calgary, Canada) and were analyzed by an independent cardiac radiologist (blinded to clinical and echocardiographic data). LV volumes (LV end-diastolic and end-systolic volumes), LVEF, and LV mass were estimated from short-axis cine images. The LVMI was calculated as the LV mass divided by the body surface area. LV strain analysis was conducted via CMR tissue-tracking (TT), which uses a 'mid-surface curvilinear coordinate system' to track myocardial deformation and follows the motion of software-generated myocardial nodes on SSFP cine sequences [1,2]. Endocardial and epicardial borders were traced in a semi-automated fashion, with manual correction for contours with apparent deviation. Using long-axis cine-images, the whole myocardial GLS

was calculated throughout the cardiac cycle by the software. As with TTE-derived GLS values, the peak negative value (peak systolic strain) was converted to an absolute |x| value, designated as the CMR-TT GLS [3]. LGE mass was measured using the full-width at half-maximum technique, as previously reported [3,4]. The total LGE mass was calculated as the summation of the LGE mass of all sections. The relative extent of the total LGE mass was expressed as a percentage of the total LV mass. Extensive LGE was defined as an LGE extent >15% of the LV mass, which has been reported to be associated with an increased risk of sudden cardiac death [5].

S1 Appendix references

[1] Hor KN, Baumann R, Pedrizzetti G, Tonti G, Gottliebson WM, Taylor M, et al. Magnetic resonance derived myocardial strain assessment using feature tracking. *J Vis Exp* 2011;12:2356.

[2] Bistoquet A, Oshinski J, Skrinjar O. Left ventricular deformation recovery from cine MRI using an incompressible model. *IEEE Trans Med Imaging* 2007;26:1136-1153.

[3] Yoon YE, Kang SH, Choi HM, Jeong S, Sung JM, Lee SE, et al. Prediction of infarct size and adverse cardiac outcomes by tissue tracking-cardiac magnetic resonance imaging in ST-segment elevation myocardial infarction. *Eur Radiol* 2018;28:3454-3463.

[4] Schulz-Menger J, Bluemke DA, Bremerich J, Flamm SD, Fogel MA, Friedrich MG, et al. Standardized image interpretation and post processing in cardiovascular magnetic resonance. *J Cardiovasc Magn Reson* 2013;15:35.

[5] Chan RH, Maron BJ, Olivotto I, Pencina MJ, Assenza GE, Haas T, et al. Prognostic value of quantitative contrast-enhanced cardiovascular magnetic resonance for the evaluation of sudden death risk in patients with hypertrophic cardiomyopathy. *Circulation* 2014;130:484-495.
