# Supplementary material for: Endocardial versus whole-myocardial tracking global longitudinal strain analysis in patients with hypertrophic cardiomyopathy: A preliminary comparative study
Source: PLoS One. 2023 Jul 11;18(7):e0288421. doi: 10.1371/journal.pone.0288421 (PMC10335699; doi:10.1371/journal.pone.0288421)
Supplement: S1 Table — (PDF) [file pone.0288421.s002.pdf]

S1 Table. Echocardiographic and CMR features according to TTE-GLS discrepancy group

	Group 1 (n=34)	Group 2 (n=8)	Group 3 (n=22)	Group 4 (n=47)	p-value
TTE parameters					
LV end-diastolic volume, ml	75 (60-88)	78 (55-86)	73 (55-93)	75 (58-83)	0.925
LV end-systolic volume, ml	25 (19-32)	27 (19-32)	24 (19-29)	27 (22-32)	0.554
LV ejection fraction, %	65 (63-68)	65 (63-67)	68 (66-72)	64 (58-68)	0.017
LV ejection fraction ≥50%	34 (100)	8 (100)	22 (100)	46 (97.9)	0.712
Maximal wall thickness, mm	17 (16-19)	19.5 (15.5-21.0)	17.5 (17.0-20.0)	18.0 (17.0-21.5)	0.148
E velocity, m/s	0.6 (0.6-0.8)	0.6 (0.6-0.7)	0.6 (0.5-0.6)	0.6 (0.5-0.7)	0.417
A velocity, m/s	0.8 (0.6-0.9)	0.7 (0.6-0.8)	0.7 (0.6-0.8)	0.8 (0.5-0.9)	0.443
s' velocity, cm/s	7.8 (6.7-8.6)	6.2 (5.3-7.2)	7.6 (6.6-8.2)	6.4 (5.2-7.4)	0.001
e' velocity, cm/s	5.5 (4.6-6.4)	5.0 (4.0-6.7)	5.1 (4.0-6.8)	4.6 (4.0-5.5)	0.054
a' velocity, cm/s	8.1 (7.0-10.1)	6.4 (5.6-8.1)	8.0 (6.5-9.8)	7.0 (6.2-8.1)	0.003
E/e' ratio	11.9 (9.5-15.4)	12.0 (9.3-16.1)	11.6 (8.2-15.0)	13.7 (11.0-16.0)	0.113
Whole myocardial GLS, %	16.9 (14.7-18.8)	12.4 (10.9-12.9)	15.3 (-16.5--14.5)	10.7 (-12.0--9.7)	<0.001
Endocardial GLS, %	24.3 (21.7-25.6)	21.4 (21.1-22.5)	19.2 (16.3-20.0)	16.6 (14.6-18.2)	<0.001
CMR parameters					
LV end-diastolic volume, ml	110 (97-123)	127 (113-147)	106 (90-122)	123 (93-127)	0.246
LV end-systolic volume, ml	34 (28-44)	46 (33-58)	32 (24-39)	33 (25-43)	0.290
LV ejection fraction, %	69 (61-77)	60 (59-75)	70 (66-72)	68 (64-75)	0.748
LV ejection fraction ≥50%	33 (97.1)	8 (100)	22 (100)	46 (97.9)	0.843
LV mass index, g/m^2	63 (43-79)	75 (71-100)	59 (54-65)	95 (71-107)	<0.001
Tissue tracking GLS, %	12.5 (11.0-15.5)	10.8 (8.0-12.7)	11.9 (10.9-13.6)	9.8 (7.8-10.8)	<0.001
LGE mass, g	3.0 (1.6-4.9)	12.8 (5.4-17.5)	5.2 (2.6-11.2)	12.8 (4.1-27.2)	<0.001
LGE extent, %	3.0 (1.6-5.2)	9.8 (4.0-14.4)	5.4 (2.4-8.5)	7.5 (2.6-14.6)	0.012
Extensive LGE (> 15% of LV mass), n (%)	1 (2.9)	1 (12.5)	2 (9.1)	12 (25.5)	0.023

Data are presented as the mean±standard deviation for continuous variables and number (percentage) for categorical variables

Abbreviations: CMR, cardiovascular magnetic resonance imaging; GLS, global longitudinal strain; LGE, late gadolinium enhancement; LV, left ventricle; TTE, transthoracic echocardiography
