# Supplementary material for: Endocardial versus whole-myocardial tracking global longitudinal strain analysis in patients with hypertrophic cardiomyopathy: A preliminary comparative study
Source: PLoS One. 2023 Jul 11;18(7):e0288421. doi: 10.1371/journal.pone.0288421 (PMC10335699; doi:10.1371/journal.pone.0288421)

S1 Fig. Plots of the correlation between each evaluated LV GLS parameter and LGE extent.

CMR, cardiac magnetic resonance imaging; GLS, global longitudinal strain; LGE, late gadolinium enhancement; LV, left ventricle; TT, tissue tracking; TTE, transthoracic echocardiography

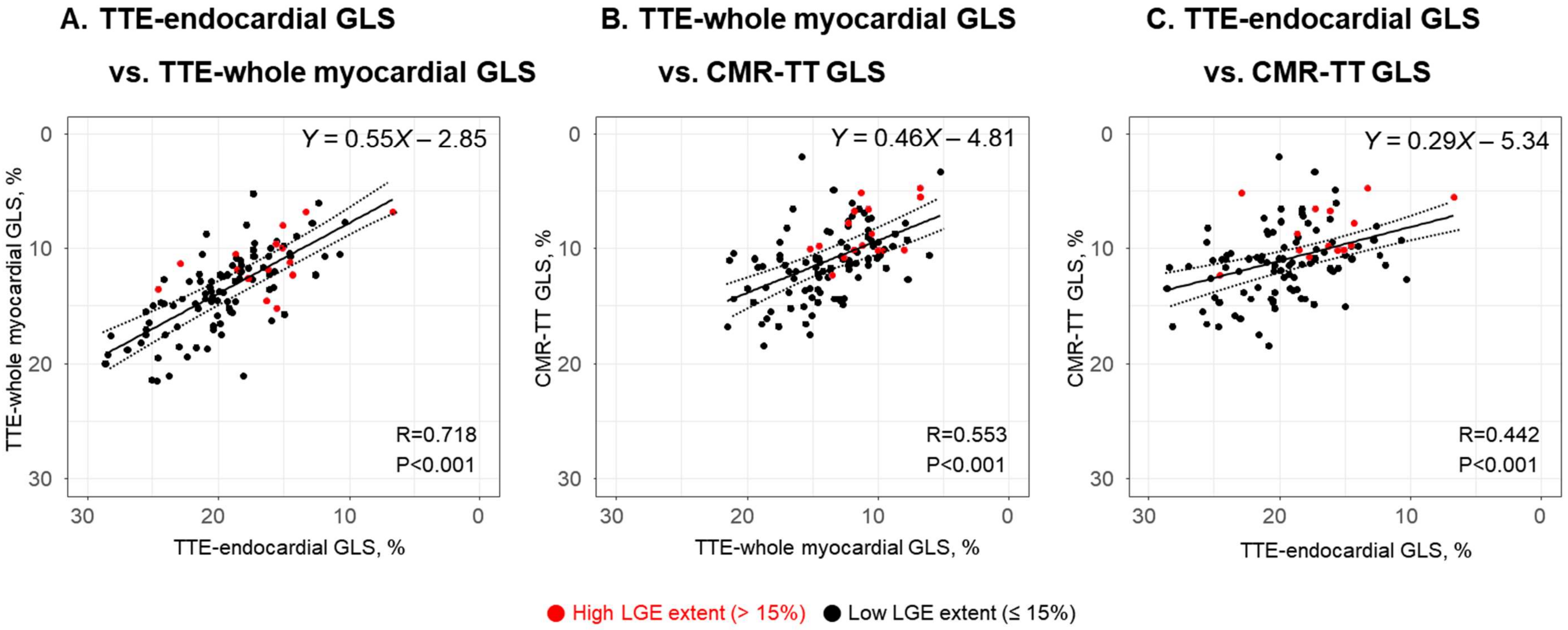

Supplement: S1 Fig — (PDF) [file pone.0288421.s003.pdf]
