# Supplementary material for: Endocardial versus whole-myocardial tracking global longitudinal strain analysis in patients with hypertrophic cardiomyopathy: A preliminary comparative study
Source: PLoS One. 2023 Jul 11;18(7):e0288421. doi: 10.1371/journal.pone.0288421 (PMC10335699; doi:10.1371/journal.pone.0288421)

S2 Fig. Bland-Altman plots of the three evaluated LV GLS parameters

CMR, cardiac magnetic resonance imaging; GLS, global longitudinal strain; LGE, late gadolinium enhancement; LV, left ventricle; SD, standard deviation; TT, tissue tracking; TTE, transthoracic echocardiography

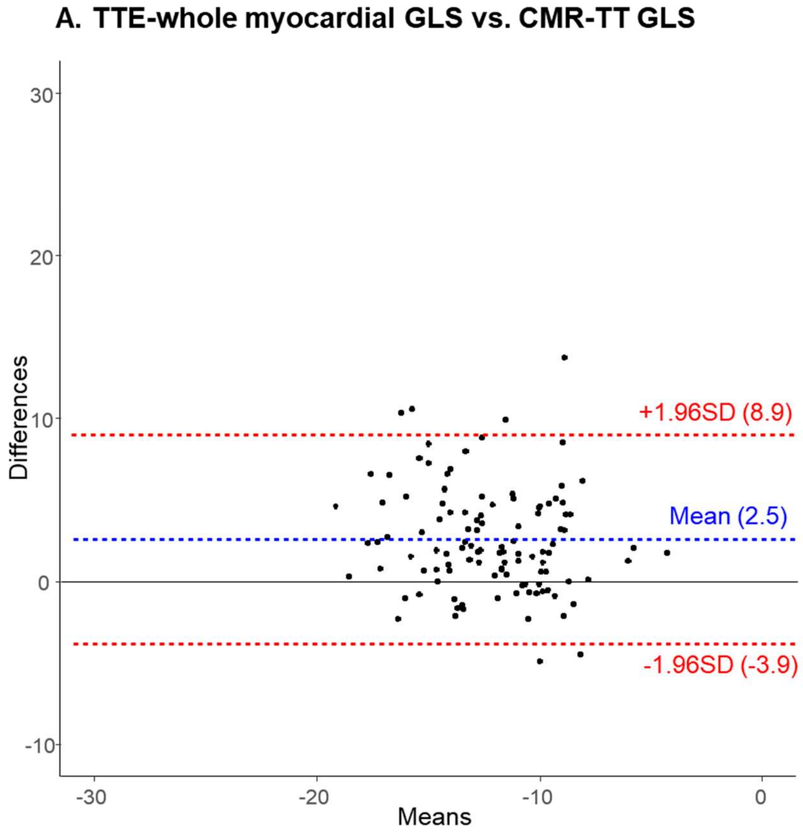

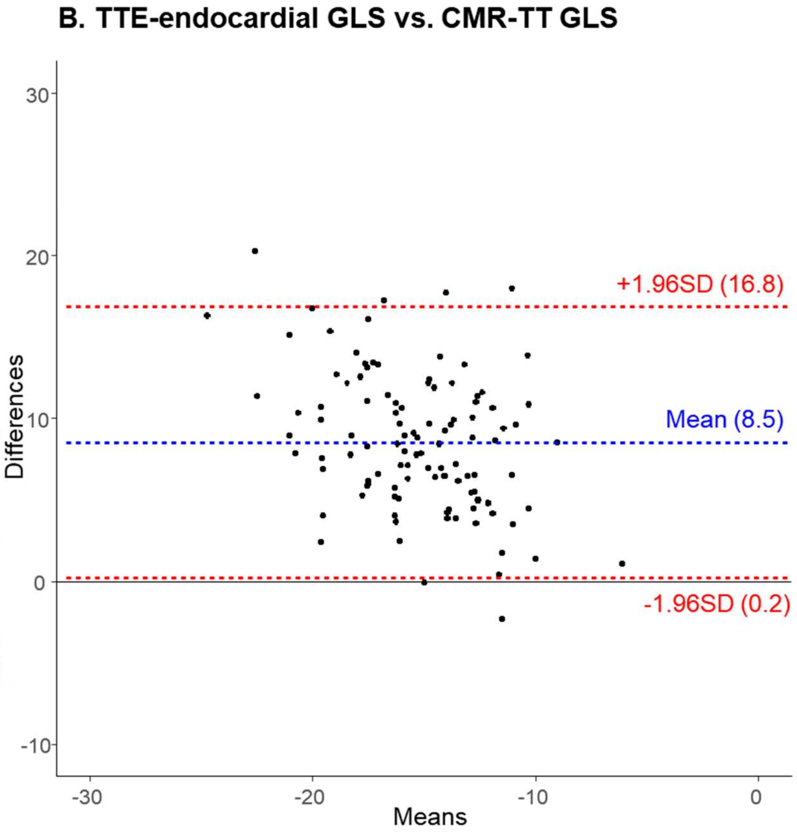

Supplement: S2 Fig — (PDF) [file pone.0288421.s004.pdf]
