# Supplementary material for: Endocardial versus whole-myocardial tracking global longitudinal strain analysis in patients with hypertrophic cardiomyopathy: A preliminary comparative study
Source: PLoS One. 2023 Jul 11;18(7):e0288421. doi: 10.1371/journal.pone.0288421 (PMC10335699; doi:10.1371/journal.pone.0288421)
Supplement: S3 Fig — (PDF) [file pone.0288421.s005.pdf]

S3 Fig. Discrimination performance of GLS parameters for extensive LGE

AUROC, area under the receiver operating characteristic curve; CMR, cardiac magnetic resonance imaging; GLS, global longitudinal strain; LGE, late gadolinium enhancement; LVMI, left ventricular mass index; TT, tissue tracking; TTE, transthoracic echocardiography

A. Total population

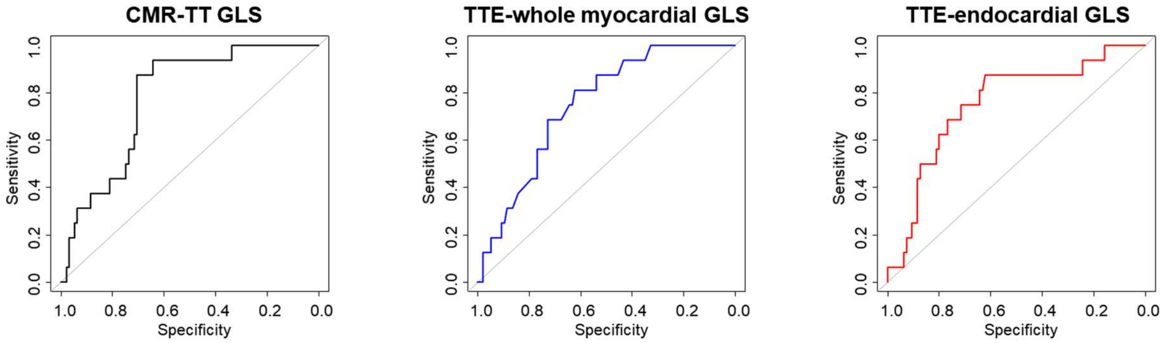

	AUROC	p for difference (vs. CMR-TT GLS)	p for difference (vs. TTE-whole myocardial GLS)
**CMR-TT GLS**	0.781 (0.762-0.799)		
**TTE-whole myocardial GLS**	0.747 (0.727-0.767)	0.001	
**TTE-endocardial GLS**	0.754 (0.729-0.779)	0.027	0.610

B. High LVMI subgroup

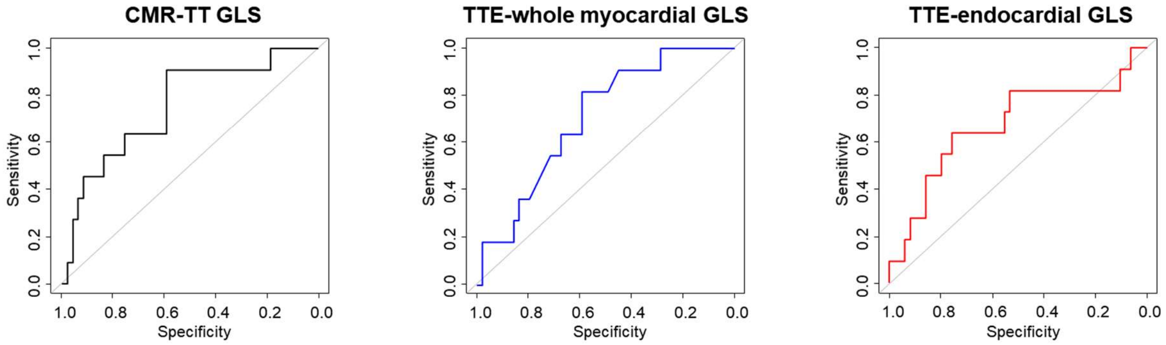

	AUROC	p for difference (vs. CMR-TT GLS)	p for difference (vs. TTE-whole myocardial GLS)
**CMR-TT GLS**	0.755 (0.728-0.783)		
**TTE-whole myocardial GLS**	0.705 (0.679-0.731)	<0.001	
**TTE-endocardial GLS**	0.668 (0.634-0.701)	<0.001	0.006
