# Supplementary figures and images for: Endocardial versus whole-myocardial tracking global longitudinal strain analysis in patients with hypertrophic cardiomyopathy: A preliminary comparative study
Source: PLoS One. 2023 Jul 11;18(7):e0288421. doi: 10.1371/journal.pone.0288421 (PMC10335699; doi:10.1371/journal.pone.0288421)

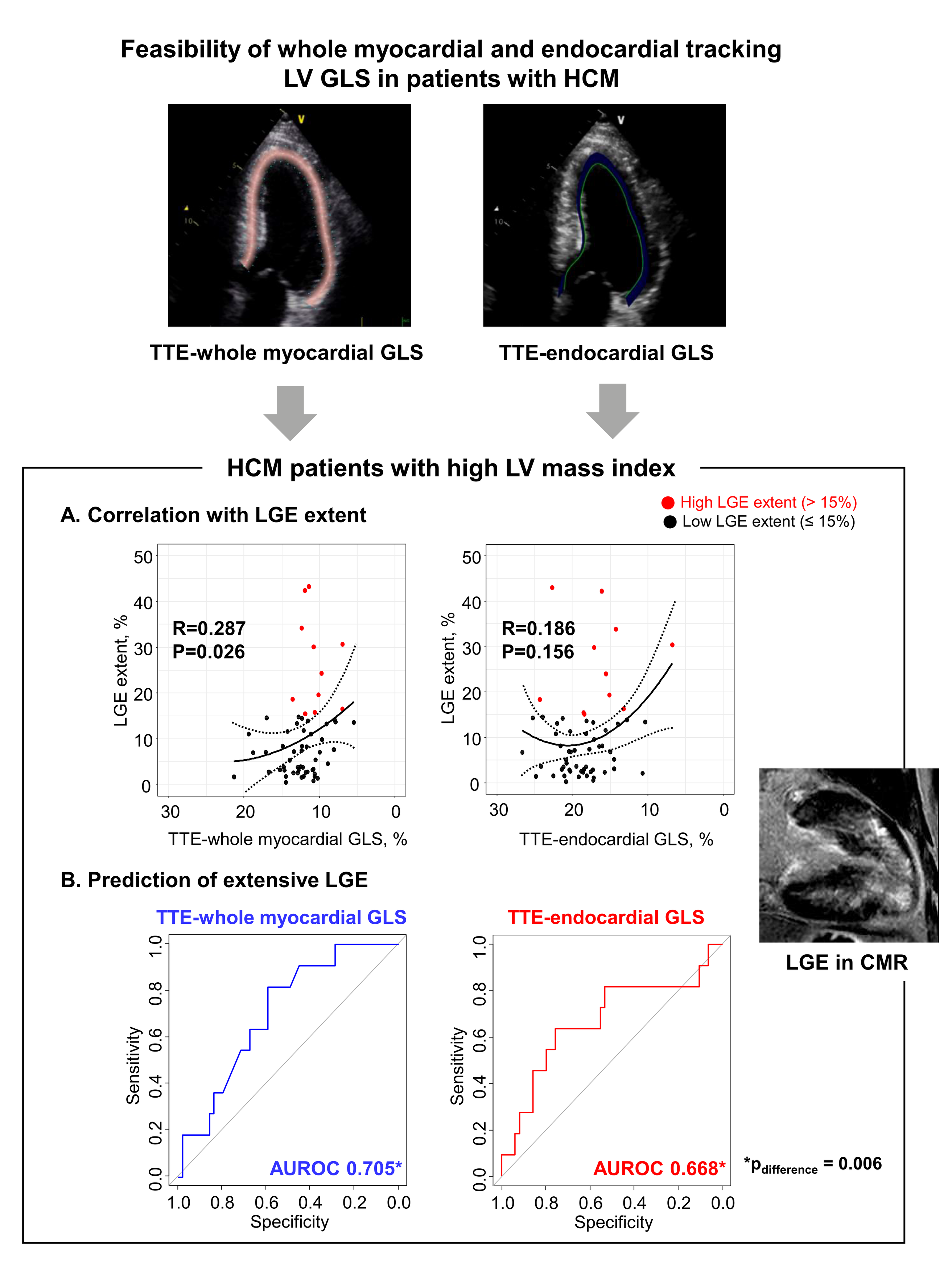

Supplement: S1 Graphical abstract — (TIF) [file pone.0288421.s006.tif]
